# Atypical cortical networks in children at high-genetic risk of psychiatric and neurodevelopmental disorders

**DOI:** 10.1038/s41386-023-01628-x

**Published:** 2023-07-04

**Authors:** Joanne L. Doherty, Adam C. Cunningham, Samuel J. R. A. Chawner, Hayley M. Moss, Diana C. Dima, David E. J. Linden, Michael J. Owen, Marianne B. M. van den Bree, Krish D. Singh

**Affiliations:** 1 Centre for Neuropsychiatric Genetics and Genomics, Division of Psychological Medicine and Clinical Neurosciences, School of Medicine, Cardiff University, Cardiff, UK; 2 Cardiff University’s Brain Research Imaging Centre (CUBRIC), School of Psychology, College of Biomedical and Life Sciences, Cardiff University, Cardiff, UK

## Abstract

Although many genetic risk factors for psychiatric and neurodevelopmental disorders have been identified, the neurobiological route from genetic risk to neuropsychiatric outcome remains unclear. 22q11.2 deletion syndrome (22q11.2DS) is a copy number variant (CNV) syndrome associated with high rates of neurodevelopmental and psychiatric disorders including autism spectrum disorder (ASD), attention deficit hyperactivity disorder (ADHD) and schizophrenia. Alterations in neural integration and cortical connectivity have been linked to the spectrum of neuropsychiatric disorders seen in 22q11.2DS and may be a mechanism by which the CNV acts to increase risk. In this study, magnetoencephalography (MEG) was used to investigate electrophysiological markers of local and global network function in 34 children with 22q11.2DS and 25 controls aged 10–17 years old. Resting-state oscillatory activity and functional connectivity across six frequency bands were compared between groups. Regression analyses were used to explore the relationships between these measures, neurodevelopmental symptoms and IQ. Children with 22q11.2DS had altered network activity and connectivity in high and low frequency bands, reflecting modified local and long-range cortical circuitry. Alpha and theta band connectivity were negatively associated with ASD symptoms while frontal high frequency (gamma band) activity was positively associated with ASD symptoms. Alpha band activity was positively associated with cognitive ability. These findings suggest that haploinsufficiency at the 22q11.2 locus impacts short and long-range cortical circuits, which could be a mechanism underlying neurodevelopmental and psychiatric vulnerability in this high-risk group.

## Introduction

Copy number variants (CNVs) are submicroscopic alterations (such as deletions or duplications) of segments of chromosomes, which constitute a major source of variation between individuals. CNVs can result in chromosomes having too many or too few dosagesensitive genes. These changes may be advantageous, indeed CNVs are thought to have been important in human evolution [1]; however, they may also have a negative impact on human development [2].

22q11.2 deletion syndrome (22q11.2DS), also known as velocardiofacial syndrome (VCFS) and Di George syndrome, is one of the most common copy number deletion syndromes, affecting at least 1 in 4000 live births [3–5]. It results from a 1.5– 3Mb deletion at region q11.2 on chromosome 22. As with many CNV syndromes, the 22q11.2DS phenotype is extremely variable, involving multiple organ systems [6]. The mechanisms underlying the pleiotropy and phenotypic variability seen in 22q11.2DS are not well understood. Neurodevelopmental, behavioural, emotional, cognitive and psychiatric problems are very common in 22q11.2DS [7–10]. The 22q11.2 International Consortium on Brain and Behavior study of over 1400 individuals with 22q11.2DS found that 21% of children with the deletion have ASD, 32% have ADHD and up to 30% develop schizophrenia in adulthood [11].

The 22q11.2 region contains approximately 60 genes but, as yet, no gene or combination of genes has been found to be either necessary or sufficient for the neurodevelopmental phenotypes observed in the deletion syndrome. It has been proposed that 22q11.2DS and other neurodevelopmental CNVs increase risk of psychopathology by affecting neuronal integration with downstream effects on local and long-range cortical networks [12]. There is mounting evidence from animal models for abnormal neuronal structure, function and migration [13–15], as well as abnormal cortical network activity in 22q11.2DS [16, 17]. In humans, postmortem studies have also found abnormal neuronal morphology and migration in 22q11.2DS [13–15, 18, 19], while structural magnetic resonance imaging (MRI) studies report reductions in cortical surface area [20], subcortical volumes [21] and white matter diffusivity [22]. Furthermore, resting-state functional MRI (fMRI) studies have found alterations across several brain networks including the default mode, sensorimotor, visuospatial, self-referential and visual networks [23–27]. Alterations in neurotransmitter concentrations have also been reported in adults with 22q11.2DS and these have been associated with psychotic symptoms [28, 29].

Synchronous oscillatory activity in the cortex reflects neuronal integration and is thought to facilitate communication within and between brain regions [30, 31]. It can be measured non-invasively in humans using EEG and MEG. Atypical oscillatory patterns have been observed in idiopathic ASD [32–34], ADHD [35–37] and schizophrenia [38–40]. Despite the proposed neuronal integration deficits in 22q11.2DS and other neurodevelopmental CNVs, there have been relatively few studies of cortical oscillations in these high-risk groups. In a recent resting-state MEG study of adult CNV carriers, we found common patterns of oscillatory dysconnectivity across nine different neurodevelopmental CNV loci, including 22q11.2DS, suggesting a putative mechanism by which neurodevelopmental CNVs could increase risk of psychopathology [41]. Due to the relatively small numbers of participants with each CNV and the high rates of psychotropic medication use, it was not possible to explore the associations between oscillatory activity and neurodevelopmental symptoms or psychopathology. To our knowledge, there have been no previous studies of resting-state cortical oscillations in children with 22q11.2DS. In the present study, we focused exclusively on children aged between 10–17 years old, to investigate the impact of 22q11.2DS on cortical network properties during this critical developmental period and to explore associations between neurodevelopmental symptoms and cognitive function. We hypothesized that children with 22q11.2 deletion syndrome would have altered oscillatory activity/connectivity and that the severity of these alterations would be associated with their cognitive and neurodevelopmental symptoms.

## Materials and Methods

### Participants

Participants were recruited through National Health Service (NHS) genetics clinics and patient support groups in the United Kingdom. 39 children aged between 10–17 years old with a diagnosis of 22q11.2DS and 26 siblings of children with neurodevelopmental CNVs (controls) were recruited to the study. Written consent was obtained in accordance with The Code of Ethics of the World Medical Association (Declaration of Helsinki) and all procedures were approved by the South East Wales NHS Research Ethics Committee. Participants over the age of 16 years old with capacity to consent gave written informed consent to participate in the study. Children under the age of 16 years old or those over the age of 16 who lacked capacity to consent for themselves gave written and/or verbal assent to take part and their parents or carers provided consent for their participation. Participants were excluded from the study if they had photosensitive epilepsy, orthodontic braces or metallic implants/prostheses in the upper half of the body. Participants taking psychotropic medication were excluded from the analyses (*n* = 1).

### Genotyping

CNV status was confirmed for all participants either by in-house testing at the Centre for Neuropsychiatric Genetics and Genomics at Cardiff University or by clinical genetics report. All control participants had inhouse microarray testing and were excluded from the analyses if they carried a neurodevelopmental CNV as defined in Kendall et al. [42] (*n* = 1). Genotyping was carried out using the Illumina HumanCoreExome whole genome SNP array, which contains 27,000 additional genetic variants at loci previously linked to neurodevelopmental disorders, including CNVs.

### Cognition and psychopathology

Psychiatric and cognitive data were collected from participating children and their primary caregivers by trained research psychologists, supervised by child and adolescent psychiatrists. Psychiatric interviews were conducted with the primary carer using the Child and Adolescent Psychiatric Assessment [CAPA [43]] to derive DSM-5 [44] ADHD diagnoses and symptom counts. The Social Communication Questionnaire [SCQ [45]] was completed by the primary carer and used to assess ASD symptoms. Children’s full-scale IQ scores were obtained using the Wechsler Abbreviated Scale of Intelligence [WASI [46]]. Further details about the clinical and cognitive phenotyping protocol have previously been reported [9].

### Data acquisition

Five-minute whole-head MEG recordings were acquired at a 1200 Hz sample rate using a 275-channel CTF radial gradiometer system. An additional 29 reference channels were recorded for noise cancellation purposes. Primary sensors were analyzed as synthetic third-order gradiometers [47]. Participants were seated upright in the MEG system during the recordings and were instructed to keep their eyes open and to attend to a red fixation point presented on a mean luminance background using a Mitsubishi Diamond Pro 2070 monitor (100 Hz frame rate) or PROPixx LCD projector (120 Hz frame rate). Electromagnetic coils were placed at three fiducial locations (bilateral preauricular regions and nasion) and their position relative to the MEG sensors was localized at the beginning and end of each recording. Relative head position at the beginning and end of the recording was used as a proxy measure of participant head motion.

After the recordings, MEG data were down-sampled to 600 Hz, band-pass filtered at 1–150 Hz and segmented into 2 s epochs, generating 150 trials per participant. Data were then visually inspected for gross artefacts such as motion, muscular contraction and eye movements. Trials containing such artefacts were removed from the dataset and excluded from further analysis. Participants with head motion greater than 30 mm or with greater than half of their trials containing artefacts were excluded from further analysis (*n* = 4).

After quality control, data from 34 children with 22q11.2DS (17 female) and 25 controls (10 female) remained and were included in the subsequent analyses. There were no significant between-group differences in head motion (range 0.5–15.3 mm, median = 4.65 mm (IQR = 5.4 mm) in children with 22q11.2DS; range 0.6–9.5 mm, median = 2.9 mm (IQR = 4.6 mm) in controls, *p* = 0.61) or the number of trials after artefact rejection (median= 131 (IQR = 34) in children with 22q11.2DS, median = 133 (IQR= 29) in controls, *p* = 0.22).

Where possible, T1-weighted MRI structural images were acquired either with a 3D fast spoiled gradient echo (FSPGR) sequence on a General Electric HDx MRI system (TR = 7.8 ms, TE = 3.0 ms, voxel size = 1mm isotropic) or a 3D magnetization prepared rapid acquisition gradient echo (MP-RAGE) sequence on a Siemens Prisma MAGNETOM MRI system (TR = 2.3 ms, TE = 3.06 ms voxel size = 1mm isotropic). Co-registration was performed by manually labelling the fiducial points on each participant’s MRI using the software package MRIViewer. T1 MRI data were not available for 15 participants due to MRI contraindications or poor data quality (e.g. due to participant head motion). For these participants, an appropriate MRI scan was selected from the available data by comparing the relative distances between the fiducial points for each participant and matching these with another participant’s dataset. The resulting co-registrations were visually inspected and a good quality match was achieved in all cases.

### MEG analysis: beamformer reconstruction

The analysis pipeline and statistical methods are described in detail in supplementary material and summarized in Fig. S1. Briefly, source reconstruction was performed using a linearly-constrained minimum variance (LCMV) beamformer in FieldTrip (version 20161011, www.fieldtriptoolbox.org) [48]. Beamforming was performed in each of six distinct frequency bands using conventional definitions: delta (1–4 Hz), theta (4–8 Hz), alpha (8–13 Hz), beta (13–30 Hz), low gamma (40–60 Hz) and high gamma (60–90 Hz). Beamformer weights were used to derive an estimated activity time series at each voxel on a 6mm grid and for each trial. These trial time series were concatenated to form a single time series for each grid voxel and then taken forward for both activity and connectivity analyses.

For each of the reconstructed grid positions, a measure of neural activity was derived in each frequency band by first computing the amplitude envelope of the virtual-sensor time series using the absolute value of the analytic function transform of the raw time series (using Matlab’s *hilbert* function) and then converting this to a single activity measure that summarizes how variable this envelope is over the entire resting-state run. To do this we calculated the coefficient-of-variation of the envelope (i.e., standard-deviation over time/mean over time). This normalized measure has the advantage of correcting for the known biases introduced by the sensitivity of beamformer weights to variations in the signal to noise ratio (SNR) of the data [49, 50]. This resulted in a 6mm isotropic activity map for each participant and each frequency band.

Functional connectivity was computed using the amplitude-envelope correlation (AEC) metric. This metric has previously been shown to be both robust and repeatable [51]. The analysis pipeline has previously been described [49]. First, spatial down-sampling to the 90 regions of the Automated Anatomical Labelling (AAL90) atlas was performed [52]. The temporal activity of each of these 90 sources was then orthogonalized with respect to each other in order to suppress any zero-time-lag correlation due to signal leakage [53]. Next, the amplitude (Hilbert) envelopes of each AAL90 region were extracted using the absolute of the (complex) analytical signal derived by the *hilbert* function in MATLAB. These amplitude envelopes were then down-sampled to a temporal resolution of 1s in order to study connectivity mediated by slow amplitude-envelope changes [54]. To obtain connectivity matrices, pairwise correlations were calculated between the 90 Hilbert envelopes, yielding 4005 unique correlations for each of the six frequency bands for each participant. Each of these correlation coefficients was then transformed to a variance-normalized Fisher z-statistic. This made the correlations suitable for further statistical analysis and corrected for the varying length of the final time series for each participant.

### MEG analysis: activity and connectivity component estimation

At the end of the above analysis procedures, each participant had 6 activity maps and 6 connectivity matrices (one for each frequency band). Each activity map had 5061 voxels and each connectivity map had 4005 unique connection values. In order to reduce the dimensionality of these features before statistical analyses, we used a data-driven analysis of the principal components using non-negative matrix factorization (NNMF, Matlab: *nnmf*). Recently, non-negative matrix factorization has been successfully used to show cohort differences in a MEG study of schizophrenia [55] and in comparing structural and functional connectivity components in healthy individuals [56]. We iteratively increased the number of components and tested what proportion of our cohort had non-zero values. We required each component to be represented in at least 50% of our participants and for the mean number of participants represented to be at least 70%. For each of the final components identified, we projected each individual’s data on to these networks to get a single component ‘strength’ for each person. For each of the 12 metrics available for each person (6 activity and 6 connectivity), we performed NNMF separately. As shown in Figs. S2 and S3, each participant’s combined activity and connectivity profile, across all 6 bands, was effectively summarized by just 79 values. It is these values that were taken forward for statistical analysis.

### Statistical analyses of NNMF derived component scores

Each of the component weightings described above was used in an analysis to determine whether their magnitude was predicted by a set of exploratory variables consisting of group status (22q11DS or control), IQ, SCQ score and ADHD symptoms. Due to the different IQ distributions in the two groups, associations with IQ were explored in each group separately. Associations with symptom scores were explored in the 22q11.2DS group only. This analysis was done by a set of univariate robust general linear modelling tests, using Matlab’s *fitlm* function. In each linear-model fit age, sex and number of MEG trials were included in the models as covariates. For linear models exploring the associations with SCQ scores and ADHD symptoms, IQ was included as an additional covariate. For each test, we assessed the significance of the principal variable in explaining variance in the residuals. Effect sizes for the principal variable of interest were calculated using standardized beta parameters and assessed for significance using p-values and 95% confidence intervals. Bonferroni correction was applied for both the number of components and the number of frequency bands tested, separately for each measure (i.e. activity or connectivity). To control for relatedness between sibling pairs in the sample, an additional sensitivity analysis using linear mixed modelling was run in R Studio (Version 1.1.383 for Mac) for each of the significant components using the package lmerTest. In these analyses, component weighting was the variable of interest with group status, age, sex and number of MEG trials as fixed effects and family identification numbers as random effects.

### Univariate whole-brain analyses of source activity and connectivity

As well as the NNMF analysis described above, we also performed mass univariate statistical analyses of the activity, at the voxel-level, and connectivity, at the edge-level. The same robust linear models were applied as in the NNMF group tests and regression, but here were applied to activity at each voxel and connectivity at each edge. To correct for multiple comparisons across the brain and across frequencies, false discovery rate (FDR) correction was used separately for activity and connectivity.

## Results

### Participant characteristics

Table 1 shows demographic, cognitive and clinical characteristics of the 34 children with 22q11.2DS and 25 controls who were included in the analyses. Children with 22q11.2DS had full-scale IQ scores that were approximately 30 points lower than controls. 29.4% of children with 22q11.2DS scored above 15/40 in the SCQ, which is a commonly used screening cut-off for a likely ASD diagnosis [57]. 17.6% of children with 22q11.2DS met DSM-5 criteria for ADHD. No controls met criteria for either ASD or ADHD diagnoses.

### NNMF analyses of source activity and connectivity

**Fig. 1:**
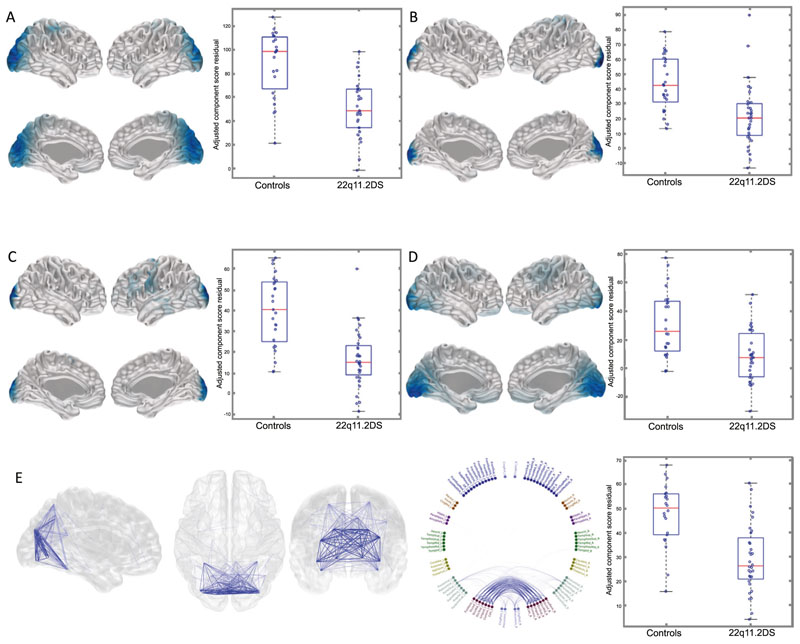
Differences in NNMF component source activity and functional connectivity between children with 22q11.2DS and controls. NNMF components showing significant differences in oscillatory activity (**A**–**D**) and connectivity (**E**) between children with 22q11.2DS and controls displayed on an MNI template mesh (**A**–**E**) and a connectivity chord plot of all AAL regions (**E**). Blue regions and lines depict components that are reduced in children with 22q11.2DS. Only those components with significant effects after Bonferroni correction (*p* < 0.05) across components and frequency are shown. **A** Activity component Beta 1 (effect size = −0.65, *p* = 2 × 10^−5^). **B** Activity component Beta 2 (effect size = −0.50, *p* = 0.0003). **C** Activity component Beta 6 (effect size = −0.69, *p* = 3 × 10^−6^). **D** Activity component Gamma (60−90) 5 (effect size −0.49, *p* = 0.0006). **E** Connectivity component Beta 1 (effect size = −0.58, *p* = 9 × 10^−5^).

NNMF analyses identified 79 components (37 activity and 42 connectivity) across the six frequency bands that were investigated (see Figs. S2 and S3). After Bonferroni correction across all frequency bands, significant between-group differences in source activity were evident in the beta and high-gamma bands with moderate to strong effect sizes. Three beta band components and one high-gamma component were identified as having significantly lower oscillatory activity in children with 22q11.2DS compared to controls. These components were located in the occipital, parietal, sensory-motor and temporoparietal regions (Fig. 1A–D).

There were also significant between-group differences in functional connectivity. Occipital and posterior-parietal beta band connectivity were lower in children with 22q11.2DS than controls (Fig. 1E).

For both the source activity and connectivity comparisons, sensitivity analysis using a linear mixed model that included sibling relatedness did not change the results (see Table S1 in supplementary materials).

### Univariate whole-brain analyses of source activity and connectivity

A similar pattern of beta and high-gamma band activity reductions were seen using a whole-brain univariate approach. However, this analysis additionally identified reductions in parieto-occipital alpha band activity and frontotemporal delta band activity. Increased bilateral frontal gamma activity was also seen in both the low and high-gamma ranges.

**Fig. 2:**
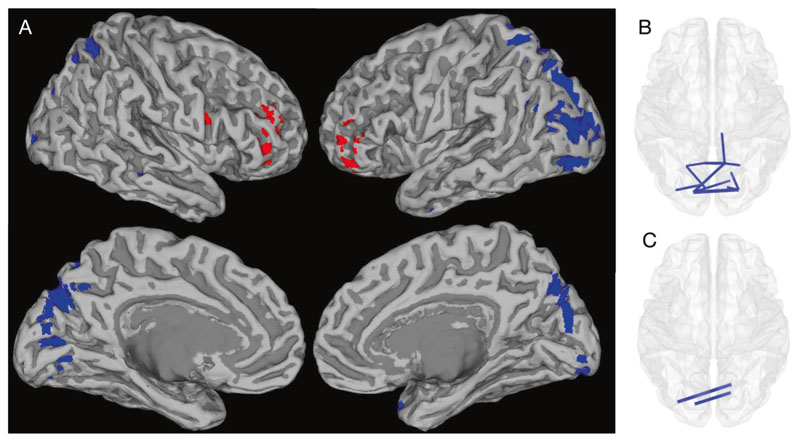
Whole-brain differences in source activity and functional connectivity between children with 22q11.2DS and controls. Whole-brain univariate analyses showing significant differences in oscillatory activity (**A**) and connectivity (**B**, **C**) between children with 22q11.2DS and controls (after FDR correction across all frequency bands) displayed on an MNI template mesh. Blue shading and lines depict effects that are reduced in children with 22q11.2DS, whilst red depicts increases in source activity. For simplicity in **A**, all voxels significant after FDR correction are shown, irrespective of frequency band (see Table S2 for details). In summary, posterior-parietal/occipital reductions in blue are in the alpha, beta and a small occipital cluster for gamma. The bilateral red hyperfrontality shown is in the two gamma frequency bands. In **B** and **C**, those connectivity edges that survive FDR correction (*p* < 0.05) are shown for the alpha and beta bands, respectively.

Figure 2 and Table S2 show the AAL atlas regions with significant source activity differences between children with 22q11.2DS and controls. Reduced alpha and beta activity were evident in occipital and parietal regions, including areas of the visual, posterior default-mode and attentional networks, whilst gamma activity was higher in the frontal lobes and lower in the occipital cortex of affected children.

As with the NNMF-based approach, the whole-brain univariate analysis identified lower occipital beta band connectivity in children with 22q11.2DS compared to controls. This approach also additionally identified significant reductions in occipital and parietal alpha band edge strength (see Table S3 and Fig. 2). Again, these posterior effects are within regions commonly considered to be within the visual, attentional and default-mode networks.

Figure 2 summarizes the spatial distribution of whole-brain effects described in Tables S2 and S3. Alpha/beta effects are confined to posterior reductions in both activity and connectivity, whilst the higher frequency gamma activity shows hyperfrontality.

### Associations with cognitive ability and neurodevelopmental symptoms

**Fig. 3:**
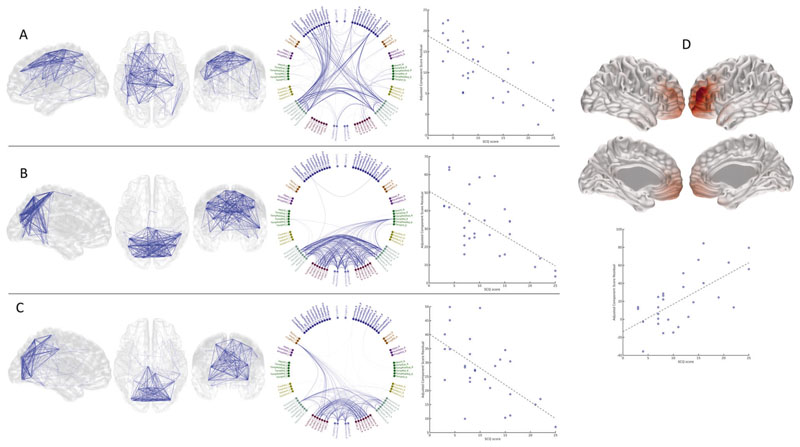
Associations between NNMF component source activity/functional connectivity and SCQ scores in children with 22q11.2DS. NNMF components showing significant associations with SCQ scores in children with 22q11.2DS (after Bonferroni correction across all components and frequency bands tested). **A** Component Alpha 2 (effect size = −0.68, *p* = 0.001). **B** Component Alpha 4 (effect size = −0.74, *p* = 0.0005). **C** Component Theta 6 (effect size = −0.85, *p* = 9 × 10^−5^). **D** Component Gamma (40-60) 1 (effect size 0.77, *p* = 0.0004). In **A**–**C**, blue lines reflect significant negative component associations with SCQ, whilst in **D**, shaded red regions show positive associations between gamma-band activity and SCQ score.

There was a significant relationship between cortical oscillatory activity and ASD symptoms in children with 22q11.2DS, with the severity of social communication difficulties being positively associated with frontal low-gamma activity and negatively associated with alpha and theta band functional connectivity. SCQ scores were associated with altered connectivity in NNMF components comprising nodes of the posterior default-mode, sensorimotor and frontoparietal control networks (Fig. 3). There were no significant associations with ADHD symptoms that survived Bonferroni correction.

**Fig. 4:**
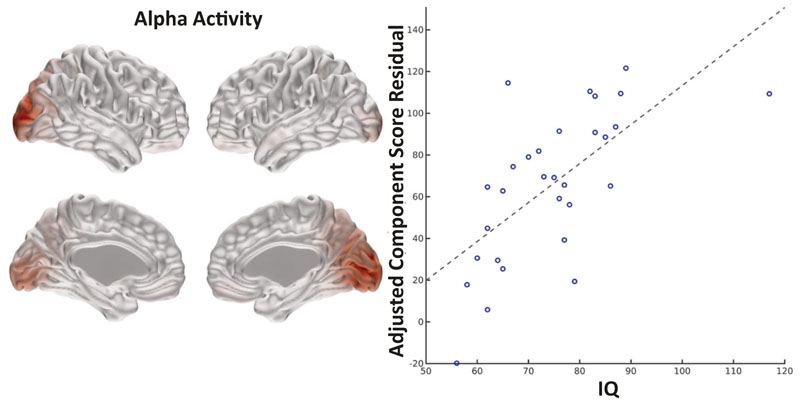
Association between alpha band source activity and IQ scores in children with 22q11.2DS. Association between NNMF component alpha 2 and IQ scores in children with 22q11.2DS (after Bonferroni correction across all frequency bands). Effect size = 0.72, *p* = 0.00058.

In children with 22q11.2DS, IQ was positively associated with alpha band activity components located in the occipital lobes (see Fig. 4). In controls, there were no significant components that survived Bonferroni correction.

## Discussion

22q11.2DS is a genetic syndrome associated with a variable and impairing neurodevelopmental phenotype. To our knowledge, this is the first study of resting-state neural oscillatory patterns in children with 22q11.2DS and the first study to explore the associations between oscillatory activity, functional connectivity and neurodevelopmental symptoms. Using robust between-group and exploratory within-group analysis strategies, we found atypical oscillatory patterns in children with 22q11.2DS, which were associated with cognitive ability and social communication difficulties. Of particular note, we found reduced low frequency oscillatory activity and connectivity in children with 22q11.2DS. These reductions were most prominent in posterior brain regions, including the occipital lobe. Reductions in low frequency activity activity/connectivity were associated with cognitive and social communication difficulties respectively. High frequency (gamma band) activity was increased in the frontal lobes (Table S2), and reduced in posterior brain regions (Fig. 1, Table S2), of children with 22q11.2DS compared with controls but interestingly, frontal gamma-band activity was positively associated with both 22q11.2DS status (Table S2) and with social communication difficulties (Fig. 3).

Our findings are broadly in keeping with previous functional imaging studies in 22q11.2DS which report alterations in the frontal, default-mode, sensorimotor and visual networks [23–27]. These networks have also been implicated in idiopathic neurodevelopmental disorders including ASD [32, 58–62] and ADHD [63–67].

High frequency oscillations in the gamma range are thought to reflect local field potentials which arise as a result of the relative balance between excitation and inhibition in local cortical circuits [68]. Cellular, animal and computational models have shown that these are largely driven by the action of parvalbumin-containing interneurons which synapse on excitatory pyramidal neurons, controlling their firing rate and synchrony [69–74]. Excitatory-inhibitory imbalance has been proposed as a common neurobiological mechanism across the spectrum of neurodevelopmental disorders seen in 22q11.2DS [35, 75–77]. There are several mechanisms by which excitatory-inhibitory balance may be perturbed in 22q11.2DS. Firstly, haploinsufficiency of *PRODH*, a gene in the 22q11.2 region, has been shown to affect GABA synthesis and gamma-band activity in murine models [78]. Secondly, these models have also found abnormal PV+ inhibitory interneuron migration in 22q11.2DS, a finding that is mirrored in postmortem studies of humans with 22q11.2DS [13–15, 18, 19]. While there is little known evidence of regional brain differences in excitatory-inhibitory balance in 22q11.2DS, it is plausible that increased frontal and decreased occipital excitability could result in pleiotropic neurodevelopmental outcomes and psychosis risk. Indeed, increased frontal and decreased occipital gamma oscillations have been reported in patients with schizophrenia [79–81].

Low frequency oscillations are thought to facilitate long-range communication between brain regions [82]. Computer simulations have shown that low frequency amplitude-envelope correlations seen in resting-state MEG studies may originate from spontaneous synchronization mechanisms in the brain [83]. Alterations in alpha, beta and theta band oscillations have been reported in idiopathic ASD, ADHD and schizophrenia [67, 77, 84–89]. We have previously found evidence of long-range (low frequency) hypoconnectivity in adults with 22q11.2DS and other neurodevelopmental CNVs [41]. 80% of participants in the adult study had a DSM-IV diagnosis (including 14% with schizophrenia) and 62% of the sample were taking medication. The relatively small sample size in that study precluded exploratory analysis of the relationships between oscillatory dynamics and clinical symptoms. In the present study, while high rates of ASD and ADHD were found, none of the children included in the analyses had a psychotic disorder and all were free from psychotropic medication. Given that up to 30% of people with 22q11.2DS develop schizophrenia during their lifetime [11], it is highly likely that a proportion of the children recruited to this study will develop psychotic symptoms in the future. Following up these children over time may yield insights into neural markers of psychosis risk.

Strengths of this study include the relatively narrow age range recruited compared to previous electrophysiological studies of CNV carriers (10-17 years), clinical and cognitive phenotyping and robust MEG analysis strategies. However, there are also a number of limitations. Due to the relative rarity of 22q11.2DS, the sample size is modest. Therefore without replication, the findings should be interpreted cautiously, due to the risks of both type 1 and type 2 error [90]. The physical health problems associated with 22q11.2DS meant that contraindications for MRI scanning were common and it was not possible to co-register MEG data to each participant’s own MRI scan. While every attempt was made to closely match the fiducial locations to those of another participant, this is not a perfect substitute for using participants’ own data and this may have affected source localization. The relatively short duration of the resting-state paradigm was chosen to balance data quantity and quality, however this will have resulted in lower signal to noise ratio than longer paradigms [91]. Unfortunately, a longer recording would not have been feasible for the majority of children taking part in this study. Furthermore, continuous head localization was not used during data acquisition so detailed analysis of head movement and correction for this was not possible. Children in both groups found it difficult to remain still during the recordings and therefore a pragmatic approach was taken to assessing data quality with higher upper limits of head motion being tolerated than in many studies involving adults (typically 10 mm). 22q11.2DS is a rare disorder associated with high rates of neurodevelopmental symptoms, particularly ADHD. We aimed to recruit a representative sample of children with 22q11.2DS, rather than one that was biased towards children with a milder phenotype. This was particularly important for investigating relationships with neurodevelopmental symptoms. After quality control, only two children had head motion exceeding 10mm and median head motion did not significantly differ between children with 22q11.2DS and controls.

While the association between frontal gamma activity, group status and clinical symptoms of ASD is interesting, this finding should be interpreted with caution as activity measures in this frequency range and in this location may be contaminated by muscle and/or eye movement artefact, which may not detectable by manual visual inspection of the data [51].

In summary, the present study investigated resting-state neural oscillatory patterns in children with a genetic syndrome associated with neurodevelopmental and psychiatric disorders. We observed alterations in oscillatory dynamics that were associated with the severity of neurodevelopmental and cognitive difficulties. These effects were seen at both high frequencies, thought to reflect local cortical processes, and at low frequencies, thought to reflect longer-range cortical communication. These results suggest a potential neural mechanism by which the 22q11.2 deletion could act to increase risk across the spectrum of neurodevelopmental disorders, involving anterior hyper- and posterior hypoconnectivity. This has implications for our understanding of 22q11.2DS, other neurodevelopmental CNVs and idiopathic neurodevelopmental disorders. In the future, longitudinal studies could shed light on how these oscillatory patterns may change during development and how they may relate to the emergence of psychotic symptoms.

## Supplementary Material

Figure S2

Figure S3

Supplementary Material

Table S2

Table S3

## Figures and Tables

**Table 1:** Participant characteristics.

	22q11.2DS	Controls	Test statistic (*t/χ*^2^)	*P*
Age, mean in years (range, SD)	13.5 (10.5–17.5, 1.9)	14.4 (10.5–17.9, 1.8)	−1.83	0.07
Gender, % female (*n*)	50 (17)	56 (14)	0.04	0.85
Handedness, % right (*n*)	76.5 (26)	76.0 (19)	<0.01	1.00
FSIQ score, mean (range, SD)	74.4 (53–117, 12.9)	107.4 (86–139, 11.0)	10.45	1.3 × 10^−14^
SCQ score, mean (range, SD)	11.0 (3–25, 6.4)	2.4 (0–9, 3.1)	6.76	1.32 × 10^−8^
Likely ASD diagnosis, % (*n*)	29.4 (10)	0 (0)	*	*
ADHD symptom score, mean (range, SD)	4.1 (0–13, 4.2)	0.3 (0–3, 0.7)	5.33	5.42 × 10^−6^
Likely ADHD diagnosis, % (*n*)	17.6 (6)	0 (0)	*	*

* Unable to calculate test statistic (*χ*^2^) or *p* value due to zero cell count in controls.

## Data Availability

Due to ethical concerns, supporting data cannot be made openly available.
