# Supplementary figures and images for: Atypical cortical networks in children at high-genetic risk of psychiatric and neurodevelopmental disorders
Source: Neuropsychopharmacology. Author manuscript; Available in PMC 2024 Jan 1. (PMC7615386; doi:10.1038/s41386-023-01628-x)

Activity

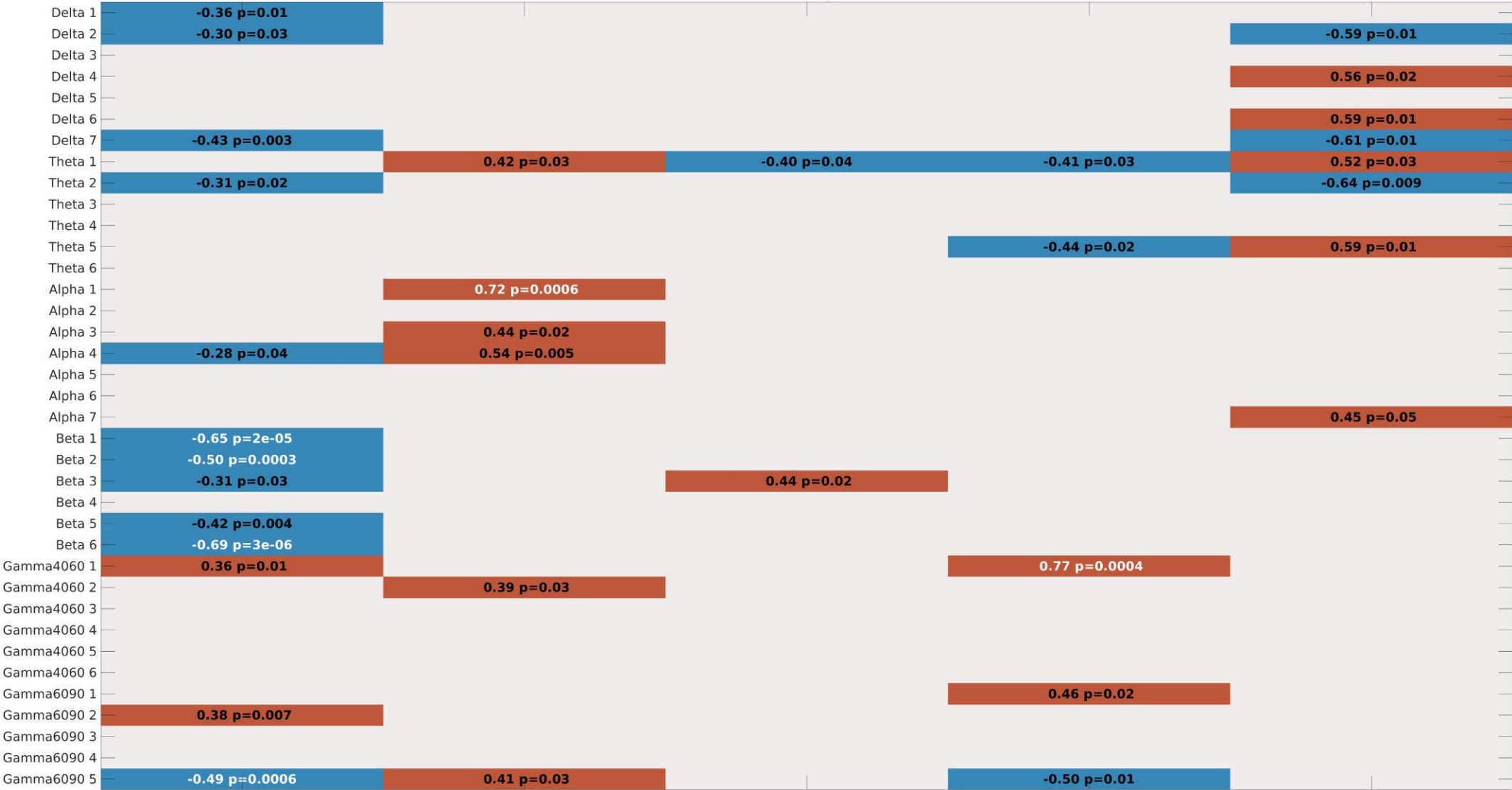

22q11.2DS vs Control

IQ in 22q11.2DS

ADHD in 22q11.2DS

ASD in 22q11.2DS

IQ in Controls

Supplement: Figure S2 [file EMS190338-supplement-Figure_S2.pdf]

# Connectivity

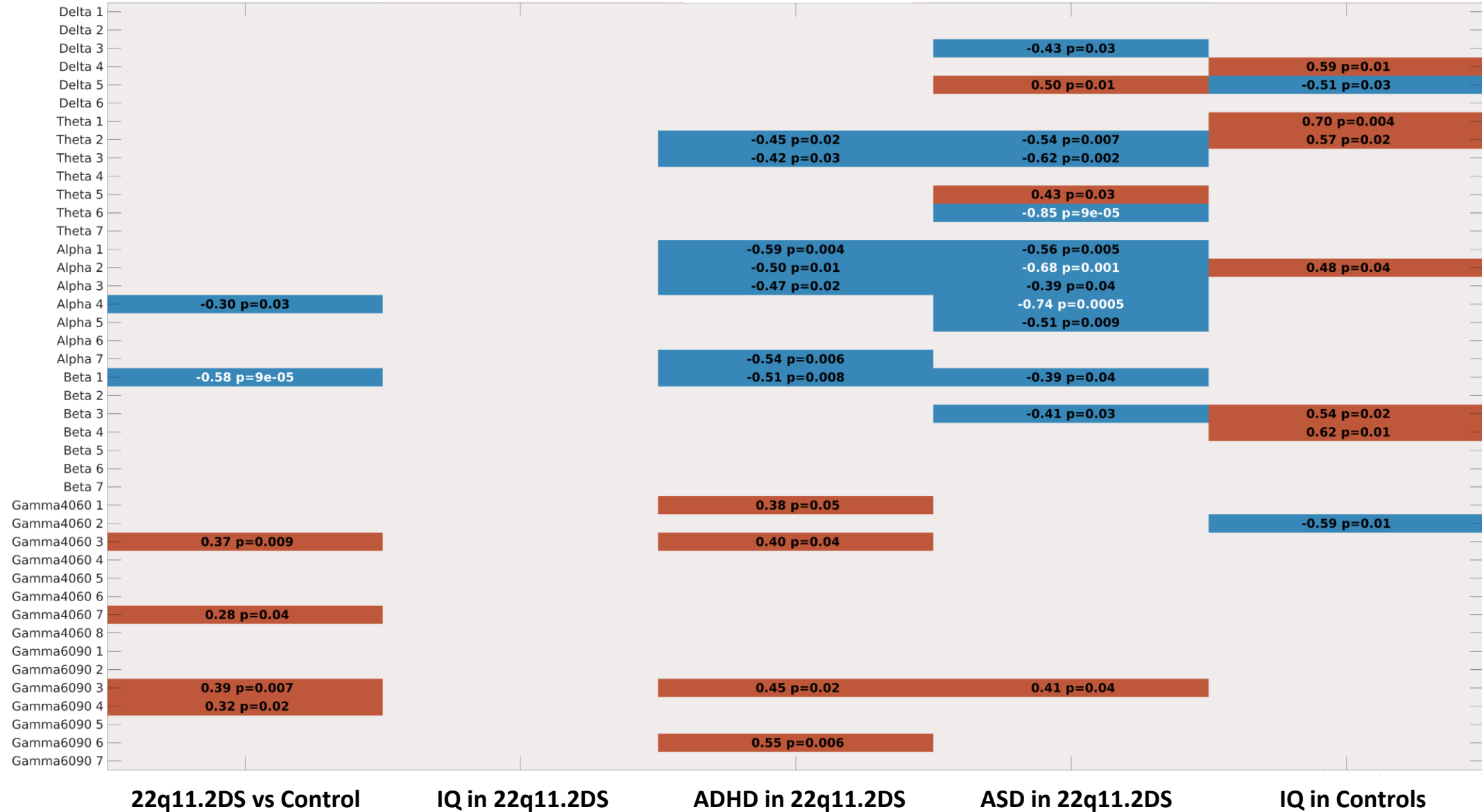

Supplement: Figure S3 [file EMS190338-supplement-Figure_S3.pdf]
