# Supplementary material for: Atypical cortical networks in children at high-genetic risk of psychiatric and neurodevelopmental disorders
Source: Neuropsychopharmacology. Author manuscript; Available in PMC 2024 Jan 1. (PMC7615386; doi:10.1038/s41386-023-01628-x)
Supplement: Supplementary Material [file EMS190338-supplement-Supplementary_Material.docx]

**Supplementary material**

Figure S1 below shows an overview of the NNMF analysis pipeline.

**Fig. S1: Overview of NNMF analysis pipeline
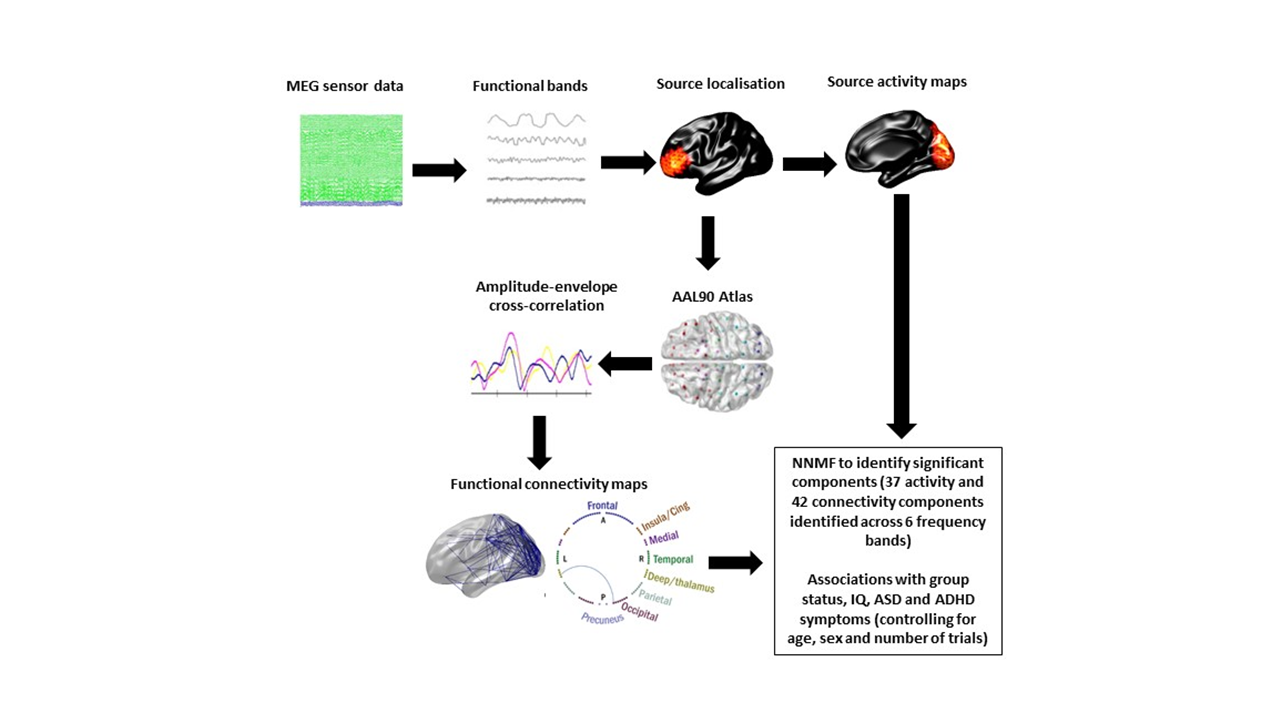
**

**Overview of the NNMF MEG analysis pipeline**. Sensor level MEG data were filtered into the six frequency bands of interest. Source localization was performed for each participant and each frequency band using a linearly-constrained minimum variance (LCMV) beamformer. Source activity was estimated for each participant and frequency band by calculating the temporal coefficient of variation (CoV) of the Hilbert envelope. For functional connectivity estimates, data were first down-sampled to the 90 regions of the Automated Anatomical Labelling (AAL90) atlas. The temporal activity of each of these 90 sources was then orthogonalized and the Hilbert envelopes of each AAL90 region were extracted. These amplitude envelopes were then down-sampled to a temporal resolution of 1s and a median spike removal filter was applied. Pairwise correlations were calculated between the 90 Hilbert envelopes, for each frequency band and each participant. Non-negative matrix factorization was performed to identify the principal components for each of the six frequency bands of interest. This reduced the data to 79 components (37 activity components and 42 connectivity components) that were taken forward for further analysis. Relationships between the components, group status, IQ and neurodevelopmental symptoms were explored using robust regression models with age, sex and number of MEG trials included in the models as covariates. As an additional whole-brain univariate analysis the same regression models were applied to voxel-level activity maps and individual edges within each connectivity matrix.

**MEG analysis: Beamformer reconstruction**

Source reconstruction was performed for each participant using a linearly-constrained minimum variance (LCMV) beamformer implemented in FieldTrip (version 20161011, www.fieldtriptoolbox.org). Beamforming was performed in each of six distinct frequency bands using conventional definitions: delta (1-4 Hz), theta (4-8Hz), alpha (8-13Hz), beta (13-30Hz), low gamma (40-60Hz) and high gamma (60-90Hz). The source-model used for reconstruction was a 6mm isotropic grid, which was initially defined in MNI template space before being matched to each individual MRI using Fieldtrip’s *ft_prepare_sourcemodel* function. The head conductivity model used was the *localspheres* option in FieldTrip, which approximates the local curvature of the head underneath each channel.

Beamformer weights were estimated for each location on the grid, via estimation of the covariance matrix over the entire resting-state recording period. After weights normalization, these weights were used to derive an estimated activity time series at each grid voxel and for each trial. These trial time series were concatenated to form a single time series for each grid voxel. After cleaning of any spike discontinuities using a temporal median filter, these time series were then taken forward for both activity and connectivity analyses.

**MEG analysis: Activity estimation**

For each of the reconstructed grid positions, a measure of activity was derived in each frequency band. This was done by first deriving the amplitude-envelope of the virtual-sensor time series using the absolute value of the analytic function transform of the raw time series (using Matlab’s *hilbert* function). The resulting time series was down-sampled to 1Hz in order to match the connectivity analysis described below and then converted to a single activity measure that summarizes how variable this envelope is over the entire resting-state run. To do this we calculated the coefficient-of-variation of the envelope, namely the temporal standard-deviation divided by the temporal mean. This normalized measure has the advantage of correcting for the known biases, introduced by the sensitivity of beamformer weights to variations in the signal to noise ratio (SNR) of the data [1,2]. The end result is a 6mm isotropic activity map, for each participant and each frequency band.

**MEG analysis: Connectivity estimation**

Functional connectivity was computed using the amplitude-envelope correlation (AEC) metric. This metric has previously been shown to be both robust and repeatable [3]. The analysis pipeline has previously been described [1]. First, spatial down-sampling to the 90 regions of the Automated Anatomical Labelling (AAL90) atlas was performed [4]. One grid source (virtual sensor) was chosen to represent each AAL90 region, based on the voxel having the largest temporal standard deviation across the resting-state experiment. The time-series of each of these 90 sources was then orthogonalized with respect to all other regions in order to suppress any zero-time-lag correlation due to signal leakage [5]. Next, the amplitude (Hilbert) envelopes of each AAL90 region were extracted using the absolute of the (complex) analytical signal derived by the *hilbert* function in MATLAB. These amplitude envelopes were then down-sampled to a temporal resolution of 1s in order to study connectivity mediated by slow amplitude envelope changes [6]. A median spike removal filter was applied to smooth large artefactual deflections in the data before further analysis.

To obtain connectivity matrices, pairwise correlations were calculated between the 90 Hilbert envelopes, yielding 4005 unique correlations for each frequency band for each participant. Each of these correlation coefficients was then transformed to a variance-normalized Fisher z-statistic, using a procedure that estimates the correlations’ null distribution for each region, using surrogates generated by randomization. This made the correlations suitable for further statistical analysis and corrected for the varying length of the final time series for each participant.

We additionally adjusted each person’s connectivity matrix, and activity maps, to correct for global session-related effects, this is common in fMRI analyses, although there is much debate as to the optimum algorithm for post-hoc standardization. Here we used a variant of z-scoring, in which the null mean and standard-deviation of connectivity values, in each person’s map, is estimated by fitting a Gaussian to the noise peak (+/- 1SD) of the distribution. These are then used to z-score each Fisher’s z-statistic of the connectivity values, for each person. A similar z-scoring procedure was also applied to the activity maps.

**MEG analysis: Activity and connectivity component estimation**

At the end of the above analysis procedures, each participant had six z-scored activity maps (one for each frequency) and six z-scored connectivity matrices. Each activity map had 5061 voxels and each connectivity map has 4005 unique connection values. In order to reduce the dimensionality of these features before statistical analyses, we used a data-driven analysis of the principal components using non-negative matrix factorization (NNMF, Matlab:nnmf). This specific algorithm was chosen because the activity measure we have used is positive-only and the slow static amplitude-connectivity measure we have used is dominated by positive correlations. For interpretability, we also preferred each participant’s loading on to each component to be a positive measure only. Recently, NNMF has been successfully used to show cohort differences in a MEG study of schizophrenia [7] and in comparing structural and functional connectivity components in healthy individuals [8].

One non-trivial issue in using NNMF is that it is difficult to decide how many components to reconstruct. Traditional stopping criteria, such as percentage variance explained, do not work well as the NNMF algorithm is able to significantly improve reconstruction accuracy by adding components that load on to small numbers of participants. We chose a heuristic approach in which we iteratively increased the number of components and tested what proportion of our cohort had non-zero values for each component. We required each component to be represented in at least 50% of our participants and, across all components, for the mean number of participants represented to be at least 70%. If, when we increased the number of components, either of these criteria was not met, we went back to the previous step. For each measure, we typically find that 5-15 components are identified to be the maximum number that meet these criteria. For each of the final components identified, we projected each individual’s data on to these networks to get a single component ‘strength’ for each person. For each of the 12 metrics we have in each person (six activity and six connectivity), we performed NNMF separately. Each participant’s combined activity and connectivity profile, across all six bands, was effectively summarised by just 79 values. It is these values that were taken forward for statistical analysis.

**Statistical analyses of NNMF derived component scores**

Each of the component weightings described above was used in an analysis to determine whether their magnitude was predicted by a set of exploratory variables consisting of group-status (22q11DS or control), IQ and neurodevelopmental symptoms, using SCQ scores and CAPA-derived ADHD symptom counts to index the severity of ASD and ADHD symptoms respectively. Due to the differing IQ distributions in the two groups, associations with IQ were explored in each group separately. This analysis was done by a set of univariate robust general linear modelling tests, using Matlab’s *fitlm* function. In each linear-model fit age, sex and number of MEG trials were included in the models as covariates. For linear models exploring the associations with ASD and ADHD symptoms, IQ was included as an additional covariate.

In each test, we assessed the significance of the principal variable (Group, ASD in 22q11DS, ADHD in 22q11DS, IQ in controls, IQ in 22q11DS) in explaining variance in the residuals after controlling for age and sex. With our relatively small participant numbers, outliers can have a strong effect on the quality of the model fit. We therefore used a form of robust fitting, using an iterative procedure, in which, after an initial fit, the residuals were assessed for outliers using Cooks’ distance. We used a common combination of rules for outlier identification: i.e. if a participant’s Cook’s distance was greater than three times the cohort mean, or had an absolute value of greater than 0.5, the participant was excluded and the linear model was re-fit. Effect-sizes for the principal variable of interest were calculated using standardized-beta parameters and assessed for significance using *p*-values and 95% confidence intervals. Bonferroni correction was applied for both the number of components tested, and the number of frequency bands within each type (activity/connectivity).

**Whole-brain univariate analyses**

As an additional analysis, the same robust linear-model fitting procedure was also applied to both voxel-wise activity maps and individual edges in the connectivity maps. As this represents hundreds/thousands of tests at the individual voxel/edge, we used the False-Discovery Rate (FDR, matlab function: *mafdr*, using the Benjamini and Hochberg method [9]).

**Results**

Figures S2 and S3 show an overview of associations between source activity/functional connectivity and group status, IQ and neurodevelopmental symptoms. Significant associations between source activity/functional connectivity and group status, IQ and neurodevelopmental symptoms for each of the six frequency bands of interest. Components (p<0.05) with positive associations are shown in red, while negative associations are shown in blue. The associations surviving Bonferroni correction are highlighted in white text. Associations are shown with effect sizes (standardized beta values) and p values. Age, gender and number of trials are included in each of the regression models. For associations with neurodevelopmental symptoms, IQ is included as an additional covariate.

To control for relatedness between sibling pairs in the sample, sensitivity analyses using linear mixed modelling (LMM) were run in R Studio (Version 1.1.383 for Mac) for each of the significant NNMF components using the package *lmerTest*. In these analyses, component weighting was the variable of interest with group status, age, sex and number of MEG trials as fixed effects and family identification numbers as random effects. The results of the LMM sensitivity analyses are shown in Table S1.

**Table S1: Resting-state activity and connectivity- linear mixed modelling results with relatedness as a covariate**

| Component | Beta Value | P |
| --- | --- | --- |
| Activity |  |  |
| Beta 1 | -0.44 | 0.00157 |
| Beta 2 | -0.48 | 0.000453 |
| Beta 6* | NA | NA |
| High gamma 5 | -0.40 | 0.00379 |
| Connectivity |  |  |
| Beta 1 | -0.56 | 0.000151 |

***** For activity component Beta 6 the sensitivity analysis could not be conducted as the mixed model failed to converge

Tables S2 and S3 show the results of the whole-brain analysis of oscillatory activity and edge connection strength differences between children with 22q11.2DS and controls. Only differences that survive FDR correction at P<0.05 are shown.

**References**

1. Koelewijn L, Lancaster TM, Linden D, Dima DC, Routley BC, Magazzini L, et al. Oscillatory hyperactivity and hyperconnectivity in young APOE-ε4 carriers and hypoconnectivity in alzheimer’s disease. Elife. 2019;8:e36011.

2. Luckhoo HT, Brookes MJ, Woolrich MW. Multi-session statistics on beamformed MEG data. Neuroimage. 2014;95:330–335.

3. Colclough GL, Woolrich MW, Tewarie PK, Brookes MJ, Quinn AJ, Smith SM. How reliable are MEG resting-state connectivity metrics? Neuroimage. 2016;138:284–293.

4. Tzourio-Mazoyer N, Landeau B, Papathanassiou D, Crivello F, Etard O, Delcroix N, et al. Automated Anatomical Labeling of Activations in SPM Using a Macroscopic Anatomical Parcellation of the MNI MRI Single-Subject Brain. Neuroimage. 2002;15:273–289.

5. Colclough GL, Brookes MJ, Smith SM, Woolrich MW. A symmetric multivariate leakage correction for MEG connectomes. Neuroimage. 2015;117:439–448.

6. Brookes MJ, Woolrich MW, Barnes GR. Measuring functional connectivity in MEG: A multivariate approach insensitive to linear source leakage. Neuroimage. 2012;63:910–920.

7. Phalen H, Coffman BA, Ghuman A, Sejdić E, Salisbury DF. Non-negative Matrix Factorization Reveals Resting-State Cortical Alpha Network Abnormalities in the First-Episode Schizophrenia Spectrum. Biol Psychiatry Cogn Neurosci Neuroimaging. 2020;5:961–970.

8. Messaritaki E, Foley S, Schiavi S, Magazzini L, Routley B, Jones DK, et al. Predicting MEG resting-state functional connectivity from microstructural information. Netw Neurosci. 2021;5:477–504.

9. Benjamini Y, Hochberg Y. Controlling the false discovery rate: A practical and powerful approach to multiple teasting. Journal of the Royal Statistical Society. 1995;57:289-300.
